# Quantifying climate feedbacks in polar regions

**DOI:** 10.1038/s41467-018-04173-0

**Published:** 2018-05-15

**Authors:** Hugues Goosse, Jennifer E. Kay, Kyle C. Armour, Alejandro Bodas-Salcedo, Helene Chepfer, David Docquier, Alexandra Jonko, Paul J. Kushner, Olivier Lecomte, François Massonnet, Hyo-Seok Park, Felix Pithan, Gunilla Svensson, Martin Vancoppenolle

**Affiliations:** 1 Earth and Life Institute, Université catholique de Louvain, Louvain-la-Neuve, B 1348 Belgium; 2 Department of Atmospheric and Oceanic Sciences, and Cooperative Institute for Research in Environmental Science, University of Colorado, Boulder, CO, 80309 USA; 3 School of Oceanography and Department of Atmospheric Sciences, University of Washington, Seattle, WA 98105 USA; 4 Met Office Hadley Centre, Exeter, EX1 3PB UK; 5 Sorbonne Université, UPMC Paris 6, LMD-IPSL, CNRS, Paris, 75005 France; 6 Earth and Environmental Sciences Division, Los Alamos National Laboratory, New Mexico, NM 87545 USA; 7 Department of Physics, University of Toronto, Toronto, M5S 1A7 Canada; 8 Earth Sciences Department, Barcelona Supercomputing Center, Barcelona, 08034 Spain; 9 Korea Institute of Geoscience and Mineral Resources, Daejeon, 34132 South Korea; 10 Alfred Wegener Institute, Helmholtz Centre for Polar and Marine Research, Bremerhaven, D-27570 Germany; 11 Department of Meteorology and Bolin Center for Climate Research, Stockholm University, Stockholm, 10691 Sweden; 12 Sorbonne Université, CNRS, IRD, MNHN, LOCEAN-IPSL, Paris, 75252 France

## Abstract

The concept of feedback is key in assessing whether a perturbation to a system is amplified or damped by mechanisms internal to the system. In polar regions, climate dynamics are controlled by both radiative and non-radiative interactions between the atmosphere, ocean, sea ice, ice sheets and land surfaces. Precisely quantifying polar feedbacks is required for a process-oriented evaluation of climate models, a clear understanding of the processes responsible for polar climate changes, and a reduction in uncertainty associated with model projections. This quantification can be performed using a simple and consistent approach that is valid for a wide range of feedbacks, offering the opportunity for more systematic feedback analyses and a better understanding of polar climate changes.

## Abstract (web summary)

Estimating the magnitude of radiative and non-radiative feedbacks is key for understanding the climate dynamics of polar regions. Here the authors propose an inclusive methodology to quantify the influence of all those feedbacks, stimulating more systematic analyses in observational and model ensembles.

## Introduction

The climate of polar regions is highly sensitive to changes in climate forcing, but also displays large internal variability. Over recent decades, northern polar regions have warmed more than twice the global average with sea-ice decreasing trends for all months of the year, especially in late summer^1–3^. In contrast, the southern polar regions have warmed less rapidly with some regions experiencing cooling and sea ice advance and others experiencing warming and sea ice loss^4–6^.

Observed changes in polar regions result from numerous interactions involving the atmosphere, land surfaces, ocean and sea ice. Due to the complexity of the underlying processes, we do not fully understand them. Advancing scientific understanding in polar regions is particularly challenging due to a short and incomplete observational record^5, 6^, large internal climate variability^6–9^ and the large biases of climate models in these regions^10^.

The feedback framework^11, 12^ offers a standard method to analyze such complex dynamics (Box 1). The first step is to define a simple reference system and to estimate the response of this reference system to a perturbation. In a second step, the internal dynamics processes are represented as feedbacks that are triggered by the initial response and amplify (positive feedback) or dampen (negative feedback) it.

In climate dynamics, the classical radiative feedback framework links global surface temperature changes to perturbations of Earth’s top-of-atmosphere energy budget^12–16^ and serves as a critical tool for quantifying climate response to greenhouse gas forcing. For instance, the magnitude of radiative feedbacks can be directly related to equilibrium climate sensitivity, commonly defined as the equilibrium global mean temperature change in response to a doubling of the CO_2_ concentration in the atmosphere^12–16^.

**Fig. 1:**
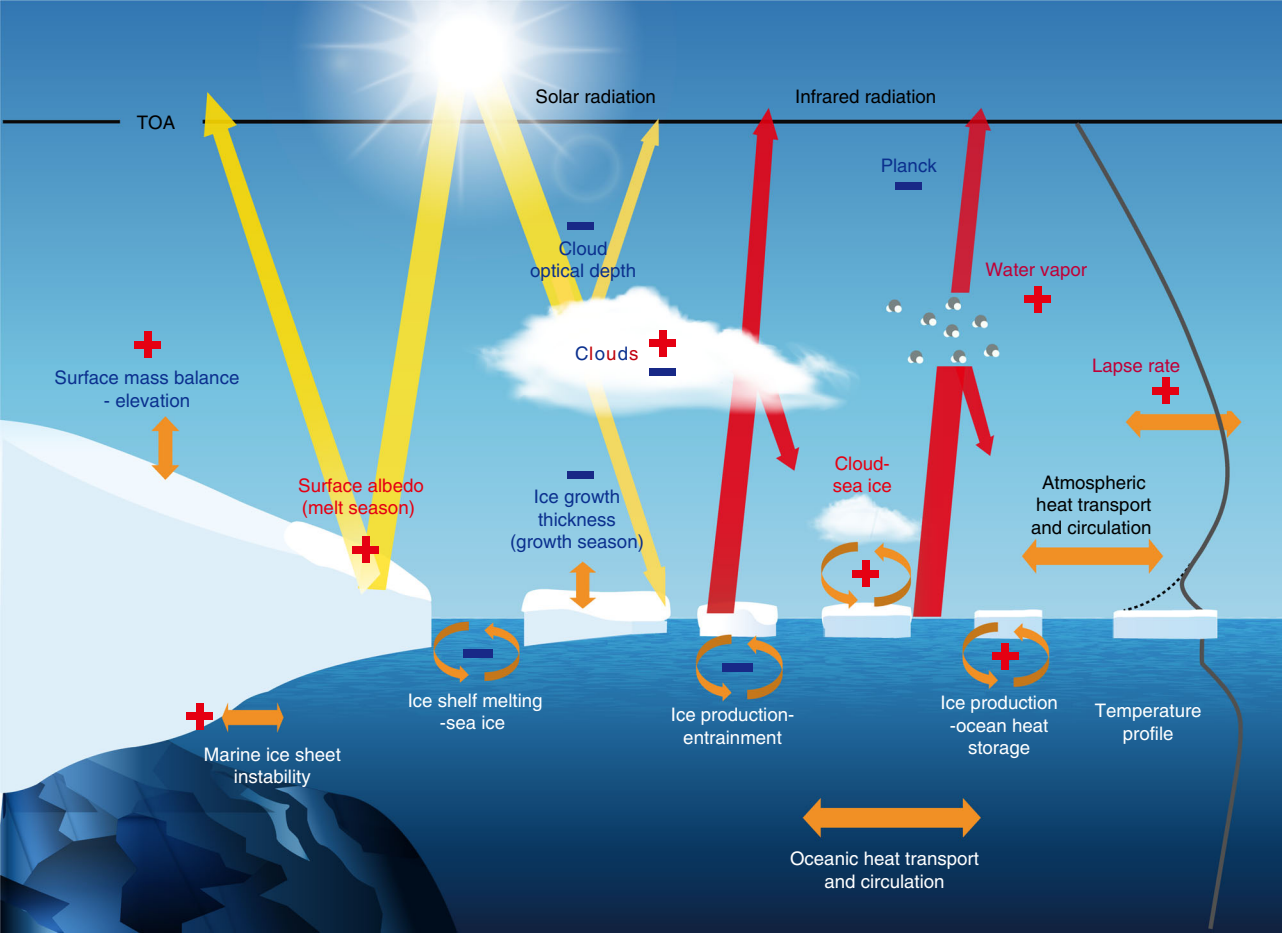
A schematic of some important radiative and non-radiative feedbacks in polar regions involving the atmosphere, the ocean, sea ice and ice sheets. TOA refers to the top of the atmosphere. Solar radiation (in yellow) and Infrared Radiation (in red) represent the shortwave (solar) and longwave (infrared) radiation exchanges. A red plus sign means that the feedback is positive, a negative blue sign corresponds to a negative feedback. Both signs are present for cloud feedbacks as both positive and negative feedbacks are occurring simultaneously and the net effect is not known. The gray line on the right represents a simplified temperature profile in polar regions for the atmosphere and the ocean, the dashed line corresponding to a strong surface inversion. Oceanic and atmospheric heat transport are mentioned but without signs as the processes involved are not restricted to polar regions and it is not clear if they could be formally expressed using a closed feedback loop

As well as radiative feedbacks, other types of feedbacks affect polar regions (Fig. 1). While analyses of radiative feedbacks in polar regions have provided clear insights into processes controlling high latitude climate change, there is much less agreement on the relative importance of non-radiative feedbacks and on the way to quantify them.

Here, we provide an overview of key radiative and non-radiative feedbacks in polar regions, how they are currently evaluated and discuss why they are important for our understanding of polar climate change. We also propose an inclusive methodology that can be applied to quantify the influence of all those feedbacks, and eventually stimulate more systematic analyses in observational and model ensembles. Estimating the magnitude of feedbacks is essential for improving our understanding of the dynamics of polar climate and to identify the relative contribution of various processes to observed high-latitude changes. In addition, it is a powerful tool to identify the origin of model biases and to reduce the uncertainty in the response to anthropogenic forcing which is directly linked to feedbacks.

### Box 1 the standard radiative feedback framework

The radiative feedback framework is based on the analysis of changes to the energy balance at the top of the atmosphere (TOA) caused by a perturbation. An initial perturbation to TOA radiation, *F* (in W m^−2^), is termed the ‘radiative forcing’ and is due, for instance, to a change in the atmospheric concentration of carbon dioxide (CO_2_) or in solar irradiance. Consider as an example the response to a positive radiative forcing resulting from an increase in greenhouse gas concentrations. This will initially lead to a decrease in outgoing longwave radiation, resulting in a TOA radiative imbalance and accumulation of energy within the climate system^117^. This in turn will trigger changes in the climate, in particular a temperature increase that leads to larger emissions of infrared radiation by the Earth. Ultimately, those larger emissions will compensate for the additional energy input due to the forcing. After some time, the climate system will come into a new equilibrium characterized by higher temperatures than before the perturbation was applied.

When studying the energy budget of the whole Earth, it is convenient to assume that the modification of the radiative fluxes emitted by the Earth is proportional to changes in global mean surface temperature *T*_s_ (in K). The imbalance of the energy budget at the TOA averaged over the whole Earth at any time (Δ*R*, in W m^−2^) is then expressed as

1 $${\mathrm{\Delta }}R = F + \lambda {\kern 1pt} {\mathrm{\Delta }}T_{\mathrm{S}}$$

where *λ* is the net climate feedback parameter (W m^−2^ K^−1^), which is a key characteristic of the climate system response, and Δ*T*_s_ is the global surface air temperature change following the perturbation. *λ* is negative for a stable climate and a larger absolute value corresponds to a less sensitive climate characterized by a smaller temperature change for a specific forcing. At equilibrium, when by definition the heat budget is balanced at the TOA (i.e., Δ*R* = 0), the surface temperature change in response to the perturbation is simply Δ*T*_s_ = −*F*/*λ*. The equilibrium climate sensitivity, estimated as the global mean temperature change in response to a doubling of the CO_2_ concentration in the atmosphere, which corresponds to a radiative forcing *F* of roughly 3.7 W m^−2^, is thus equal to −3.7/*λ*. The transient imbalance at the TOA corresponds to heat storage, which is mainly accounted for by the ocean, so the term Δ*R* is often approximated by the ocean heat uptake^66^.

The net climate feedback parameter *λ* can be separated into contributions from changes in surface albedo, clouds, water vapor and temperature, referred to as the feedback variables. The feedback related to temperature is itself the sum of a contribution from vertically homogenous warming or cooling (black-body response or Planck feedback, denoted by *λ*_0_) and one from changes in vertical temperature gradient (the lapse rate feedback). For each process, a specific feedback parameter *λ*_*i*_ can be computed. Their sum approximatively gives back the net climate feedback parameter $\lambda = \lambda _0 + \mathop {\sum}\limits_i {\lambda _i} + \varepsilon$, where *ε* is a small residual accounting for non-linearities. A positive value of the feedback parameter *λ*_*i*_ corresponds to a positive feedback, a negative one to a negative feedback
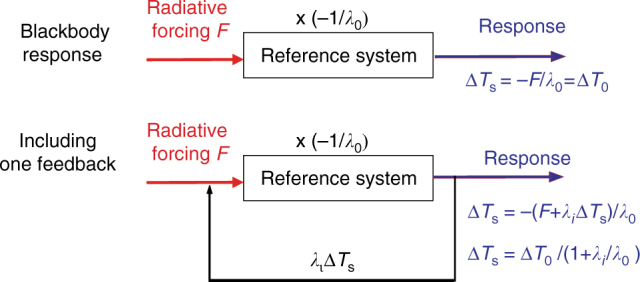


Schematic illustration of the radiative feedback framework (based on ref. ^12^). With no feedback, the reference response of the system to a radiative forcing *F* is considered to be Δ*T*_0_ = −*F*/*λ*_0_. A feedback, with a feedback parameter *λ*_*i*_, will induce a change in the radiative balance *λ*_*i*_Δ*T*_s_ that will reinforce or dampen the effect of the radiative forcing, leading to a response of the system of Δ*T*_s_ = −(*F* + *λ*_*i*_Δ*T*_s_)/*λ*_0_.

## Feedbacks in polar regions

### Radiative feedbacks

The temperature feedback represents the changes in infrared (longwave) radiative fluxes due to changes in surface and tropospheric temperatures (Table 1). It can be decomposed into a Planck feedback due to radiation changes caused by vertically uniform warming of the surface and troposphere and a lapse rate feedback due to vertically non-uniform warming^17^. The negative Planck feedback is the climate system’s basic response to forcing that drives the system to a new equilibrium temperature. Due to the dependence of blackbody radiation on temperature, the Planck feedback, or in other words the increase in outgoing longwave radiation per unit of local warming, is less negative in polar regions than at lower latitudes^18^. While the lapse rate feedback is negative in the tropics, it is often positive in polar regions because stable stratification, especially in non-summer months, suppresses vertical mixing and warming remains largely confined to a thin near-surface layer^19, 20^.

**Table 1:** Key radiative and non-radiative feedbacks in polar regions that are related to the atmosphere, ocean, sea ice, ice sheets and land surfaces and can be measured using a feedback factor

	Name	Description	Measure	Reference(s)
Radiative feedbacks	Planck (−)	Higher surface and atmospheric temperatures increase outgoing longwave radiation, avoiding runaway warming	Change of TOA flux due to temperature change at constant lapse rate divided by surface temperature change	12–14,18
	Lapse rate (+ in Arctic, close to 0 in Antarctic)	In a warmer world and at high latitudes, stable stratification conditions in the lower troposphere result in a larger warming of the lower than of the upper troposphere, leading to a smaller increase in outgoing longwave radiation compared to vertically uniform warming, and thus to further warming	Change of TOA flux due to lapse rate changes divided by surface temperature change (normalized by Planck feedback)	19,20
	Surface albedo (+)	Melting ice and snow lowers surface albedo, leading to increased absorption of shortwave radiation and amplified warming	Change of TOA flux due to surface albedo change divided by surface temperature change (normalized by Planck feedback)	19,27,28,101
	Water vapor (+)	In a warming climate, the amount of water vapor in the atmosphere increases, which amplifies the greenhouse effect and leads to further warming	Change of TOA flux due to water vapor change divided by surface temperature change (normalized by Planck feedback)	22–24
	Cloud (+/−) Two examples are provided below	Warming of the atmosphere leads to changes in the amount and characteristics of clouds, modifying the radiative balance. The cloud contribution can be decomposed in several ways, two examples being given below	Change of TOA flux due to changes in cloud properties divided by surface temperature change (normalized by Planck feedback)	14,31–41
	Example 1: Cloud-sea ice (+ in non-summer months, close to 0 in summer)	Decreased sea ice extent in non-summer months results in greater cloud cover and increased downwelling longwave radiation, leading to further sea ice loss	Change of TOA flux due to changes in cloud amount and opacity resulting from varying sea ice concentration divided by surface temperature change	36–39
	Example 2: Cloud optical depth (−)	As the climate warms, the fraction of liquid water in mixed-phase clouds increases, resulting in higher cloud albedo, more reflection of shortwave radiation and reduced warming	Change of TOA flux due to changes in cloud optical depth divided by surface temperature change	32,34,40
Non-radiative feedbacks	Ice production–entrainment (−) (mostly active in Southern Ocean)	Brine rejection during sea ice formation induces an ocean mixed layer deepening that brings to the surface warmer water from deeper levels, melting a part of the ice initially formed and inhibiting further ice production.	Ratio of the sea ice melt due to the entrainment of warmer water in the mixed layer to the initial ice formation	50,51
	Ice production–ocean heat storage (+) (mostly active in Southern Ocean)	Anomalous sea ice production induces vertical exchanges of salt, a higher stratification, storage of heat at depth and finally lower oceanic heat fluxes that favor further ice production.	Ratio of the latent heat associated to ice production to the heat content change of the ocean subsurface layer	52,53
	Ice growth–thickness (−)	Thin sea ice grows more rapidly than thick sea ice due to its higher heat conduction, dampening the response to an initial decrease imposed by a perturbation.	Normalized difference in the thickness response to an energetic perturbation with and without thickness dependence of the ice growth rate	48,49
	Surface mass balance–elevation (+) (mostly active in Greenland Ice Sheet)	Increased air temperature leads to ice melting, which lowers the surface elevation of the ice sheet, hence leading to ice exposure to warmer air temperatures and further ice melting.	Ratio of the additional sea level contribution due to this feedback to the sea level contribution without feedback	56,57
	Ice shelf melting sea ice (−) (mostly active in Southern Ocean)	Ocean warming leads to ice shelf melting, which releases freshwater into the ocean and reduces vertical mixing. This results in sea ice expansion and reduced ocean warming.	Ratio of the additional change in sea ice extent caused by this feedback to the total change in extent without feedback	63,64
	Marine ice sheet instability (+) (mostly active in West Antarctic Ice Sheet)	An initial retreat in the grounding line position of a marine ice sheet on an upward-sloping bed towards the ocean leads to increased ice discharge, ice thinning and further retreat.	Ratio of the additional sea level contribution due to this feedback to the sea level contribution without feedback	58–60

The proposed selection is illustrative rather than exhaustive. The sign in the first column indicates whether the feedback is positive or negative in polar regions

As the surface warms, additional water vapor amplifies the greenhouse effect and induces further warming^21, 22^. This water vapor feedback is largest in the tropics where the climatological temperature is higher and the increase in water vapor is at its maximum. In polar regions, the positive water vapor feedback is weaker than in the tropics but it still plays a relevant role in the polar response to the forcing^19, 23, 24^.

The surface albedo feedback is a first-order visible (shortwave) positive radiative climate feedback mechanism in polar regions^25–28^. As the climate warms, snow and ice cover melt, exposing underlying surfaces that typically have much lower albedos. This leads to an increased absorption of shortwave radiation by the surface, and as a result amplifies the initial warming. When melting, the snow covering Arctic sea ice contributes to forming melt ponds. increasing the absorption of solar radiation and amplifying the surface albedo feedback^29^. Melt ponds do not form in the Southern Ocean as surface melting is very limited there, providing an illustration of different ways snow and ice interactions affect the surface albedo feedback^29, 30^.

Clouds influence the heat balance of the Earth by affecting the radiative fluxes in both visible and infrared bands and are involved in a variety of feedbacks^14, 31, 32^. The sign of any cloud feedback depends on the balance of shortwave cooling and longwave heating by the clouds. Cloud feedbacks are the most uncertain of all the radiative feedbacks as the cloud radiative effect depends on several factors that can be modified by the initial response to the perturbation^14, 33–35^. Among all mechanisms involved, two polar-specific cloud feedback examples are listed in Table 1: the cloud sea-ice feedback^36–39^ and the cloud optical depth feedback^32, 34, 40^. When sea ice melts and new open water is exposed, surface turbulent heat fluxes can increase humidity in the lower atmosphere and increase low-level clouds. During polar night, increasing low cloud increases downwelling longwave radiation, leading to further sea ice loss and thus to a positive feedback. Observational evidence shows that this cloud-sea ice feedback operates in non-summer months in both the Arctic^37, 39^ and the Antarctic^41^. The cloud optical depth feedback operates both at mid- and high- latitudes. Cloud liquid particles are smaller than cloud ice particles, and are therefore more efficient at reflecting solar radiation back to space. As the climate warms, the total amount of cloud water in mixed phase clouds increases, which increases the amount of reflected solar radiation (i.e., increase the planetary albedo), acting as a negative feedback^32^. At the same time, the fraction of cloud water that is liquid also increases, enhancing the effect of this cloud optical depth feedback (Supplementary Fig. 1). Climate models robustly show this feedback^34^ but in a manner that is stronger than implied by observations^42, 43^ —in many cases due to excessive cloud ice in the present-day modeled climate^44–46^.

### Feedbacks related to sea ice and the ocean

As the magnitude of some radiative feedbacks is modulated by processes that may appear hidden in their overall evaluation, focused analyses have been proposed to reveal elements specifically related to sea ice and ocean. For instance, the surface albedo feedback is more efficient for thin than for thick sea ice as a similar change in thickness induced by a given perturbation will lead to a larger increase in the open water area and thus a larger change in albedo. This has led to the definition of the open water formation efficiency as the percent open water formation per cm of ice melt over the melt season^47^.

Some other feedbacks are not directly related to radiative processes. For example, basal sea ice growth rate is largely driven by heat conduction, which varies as the inverse of sea ice thickness: thin ice grows much faster than thick ice^48^. At the same time, sea ice melt rate is nearly independent of ice thickness. This leads to the negative ice growth-thickness feedback. When a positive radiative perturbation is applied to the sea ice surface energy balance leading to an initial ice thinning, ice formation in winter is enhanced so that the ice adjusts its thickness to match the new growth rates to the new melting rates^49^, resulting in a new equilibrium for the sea ice thickness. As the thermal insulation power of snow is even more efficient than that of sea ice, its influence on the ice growth-thickness feedback is important, but not fully quantified due to the complex relationship between snow depth and sea-ice thickness^30^.

Because of the large heat fluxes at the ocean–ice interface in the Southern Ocean, feedback analyses there have often focused on the interactions between ocean and sea ice. In the ice-covered parts of the Southern Ocean, the stability of the upper water column is controlled by salinity, with the ocean temperature and salinity increasing with depth below the surface mixed layer (the ocean layer that has a nearly homogeneous density). In winter, when brine is released by sea ice formation, surface mixed layer density increases, inducing a mixed layer deepening and the entrainment of relatively warm water into the surface oceanic layer. This warm water reduces ice formation and can even melt ice, which partly compensates for the initial ice formation, leading to a negative ice production–entrainment feedback^50, 51^

The ice production–entrainment feedback is acting at the seasonal scale but the magnitude of the ice–ocean flux can also be modulated at inter-annual to decadal timescales, leading to the positive ice production heat-storage feedback^52, 53^. If ice production is very large during a particular year, the mixed layer will be deeper and the salt released by ice formation will be distributed over a larger depth range. In summer, the mixing is weaker and the freshwater input due to ice melting will be spread over a shallower layer, leading to a net downward vertical salt transport. This can restrain the vertical exchanges in the water column the subsequent winter, leading to less heat transfer to the surface and more heat storage at depth. Finally, the weaker heat flux at the ice–ocean interface would favor additional ice formation, leading to a positive feedback amplifying the initial perturbation. The heat storage at depth can also be reinforced by a net freshwater input at the surface (due for instance to a net transport of sea ice to the region) that further stabilizes the water column.

### Feedbacks related to land surfaces and ice sheets

At low and mid-latitudes, a drying of the soils in response to an initial temperature rise can amplify the warming as the evapotranspiration that normally cools the surface is reduced^54^. This positive soil moisture–temperature feedback is less active at high latitudes but, despite the low temperatures, evapotranspiration strongly contributes to moderate the summer warming over land^55^.

Ice sheets, glaciers and snow cover over land provide key components to the surface albedo feedback^25–28^. They also give rise to a number of specific feedback mechanisms. Three important ones are discussed here. Compared to the feedbacks mainly involving the atmosphere and sea ice, ice sheets generally, but not exclusively, play a role on longer time scales. In the positive surface mass balance–elevation feedback, increased air temperature leads to ice melting, which lowers the surface elevation of the ice sheet, exposing the ice to warmer air temperatures and thus further melting^56, 57^. This positive feedback is mostly relevant for the Greenland ice sheet where surface melting is substantial, while currently the Antarctic ice sheet hardly experiences it due to very low surface temperature.

The marine ice sheet instability has the potential to destabilize large ice sheet regions^58–60^. The stability of a marine ice sheet such as the West Antarctic Ice Sheet is determined by the position of the grounding line, i.e., the boundary between the grounded ice sheet and the floating ice shelf. If it is located on a bedrock sloping downward toward the interior of the ice sheet, an initial retreat of the grounding line, for instance due to basal ice melting, leads to an increase in ice discharge, which results in a further retreat of the grounding line inland until a new stable position is reached. Rapid changes in ice sheets may also be linked to the acceleration of the ice transport due to basal lubrication caused by meltwater penetration at the bed or to breakup of the ice shelves because of a weakening of the ice due to surface melting^61, 62^.

Another feedback mainly acting in the Southern Ocean is related to the interactions between floating ice shelves, sea ice and the surrounding ocean. A subsurface Southern Ocean warming leads to increased basal ice shelf melting, and the upper ocean layers get fresher due to the resulting cold freshwater input. This results in lower heat flux from the ocean interior to the surface, sea ice expansion and reduced ocean surface warming, providing a negative ice shelf melting sea ice feedback^63, 64^.

### Non-local feedbacks and feedbacks involving other components of the climate system

While this Perspective focuses on feedbacks that act through physical processes in polar regions (Table 1, Fig. 1), we should mention that many other feedback processes exist, some of which involve biological processes and biogeochemical cycles^65–68^. One example is the bio-optical feedback, which occurs when climate warming and sea ice retreat in the Arctic Ocean lead to intense phytoplankton blooms^69^. These blooms trap the penetrating solar heat flux at the ocean surface, which increases sea surface temperature. As a result, sea ice concentration decreases, which leads to enhanced absorption of solar energy into the ocean and further warming of the Arctic^70^.

The response to a perturbation also implies a redistribution of the energy between different latitudes. First, the warming of the tropics under greenhouse gas forcing leads to enhanced poleward energy transport by the atmospheric circulation to higher latitudes, contributing to warming there^71–75^. This indicates a coupling between radiative feedbacks and atmospheric heat transport^74, 76, 77^. Moreover, radiative feedbacks at low latitudes may influence polar warming through their effect on poleward energy transport, while changes in polar regions may affect dynamics in the lower latitudes^78^. This is an area of ongoing research, and it is not yet clear if the response of the system has a direct impact on the original perturbation itself such that a closed feedback loop can be identified.

Second, the ocean heat transport has been found to strongly shape polar climate change, with increased poleward heat transport into the Arctic^79–81^ and decreased poleward heat transport into the Southern Ocean^4, 79^ under global warming. Here too, it is unclear whether these changes can be represented in terms of a closed feedback loop (e.g., sea ice thinning enhancing ocean heat transport into the Arctic^81^) or should be classified as important drivers of polar climate change that cannot be expressed within a feedback framework.

## Quantitative evaluation of feedbacks in polar regions

### Radiative feedbacks

The feedback parameters provide classical measures of the magnitude of the radiative feedbacks (Box 1). They are defined as the change in radiative fluxes due to the impact of a change in surface temperature upon the variable of interest (e.g., surface albedo, water vapor amount, cloud cover also referred to as climate variable) and are quantified in W m^−2^ K^−1^. The net climate feedback parameter *λ,* which is equal to the sum of all the parameters for the individual feedbacks, can be estimated by measuring all the terms of the equation describing the global mean radiative balance (Eq. (1) in Box 1) or by regressing the change in radiative flux at the top of the atmosphere (TOA) against the global mean surface temperature change^82^. It is somewhat more complex to evaluate specific feedback parameters *λ*_*i*_, as this requires isolating the impact of each feedback variable on the Earth’s energy balance^31, 33, 83–87^.

Since TOA fluxes determine the total energy budget of the Earth’s climate system, they are a natural reference point for computing climate feedbacks at a global scale. They are also closely connected to surface temperature change in the Tropics, where deep convection leads to a vertically well-mixed atmosphere. In the Arctic, where deep vertical mixing is suppressed by strong static stability in the troposphere, computing feedback parameters based on surface fluxes can lead to important additional insights^19, 23, 55, 74^. For example, a change in clouds that raises atmospheric emissivity in the Arctic inversion layer can lead to increases in both upwelling and downwelling longwave radiation, and thus lead to energy loss and a negative cloud feedback at TOA but energy gain and a positive cloud feedback at the surface^19, 88^.

Individual feedback parameters defined at the surface or TOA can be diagnosed using several different methods, including partial radiative perturbations^31^, the less computationally expensive approximate partial radiative perturbations^83^, and the even more idealized radiative kernel technique^85, 86^. Using this now widely used method, changes in TOA radiative fluxes due to a uniform, idealized perturbation in the feedback variable are first computed using a radiative transfer model to obtain the so-called kernel. The kernel thus only depends on the radiative transfer algorithm and the mean state of the system. *λ*_*i*_ can then be derived by multiplying the kernel by the response of the feedback variable to changes in global mean surface temperature.

In parallel to feedback parameters, other expressions can sometimes be easier to interpret or be more convenient. One option is to diagnose the temperature change that can be attributed to each feedback explicitly, known as a warming contribution (see the methods). It is also instructive to compare the temperature changes due to a particular feedback to changes of a reference system in which the feedbacks of interest are inactive. In the radiative feedback framework, the reference system is traditionally chosen as the Planck response. The feedback factor *γ*_*i*_ is then defined as the ratio of each feedback parameter to (minus) the Planck feedback *λ*_*0*_: *γ*_*i*_ = *λ*_*i*_/−*λ*_0_. An advantage of this approach is that the feedback factor *γ*_*i*_ is dimensionless because it is expressed relative to the reference system. It can then be used to compare the impact of very different processes, bearing in mind that its specific value depends on the reference system chosen^12^(for more details see the methods).

In addition to the approaches focused on the top of the atmosphere or the surface, it is possible to analyze the origin of three-dimensional temperature changes such as in the climate feedback response analysis method^89^ (CFRAM). It has also been proposed to decompose the feedbacks in ways that differ^55, 90^ from the traditional one described in Box 1. Each methodology is adapted to a special purpose but also has its own limitations. For instance, a three-dimensional analysis can highlight the processes that are at the origin of the changes at various level in the atmosphere, but it may require model outputs that are not routinely saved by climate modeling centers. Finally, applying different methods leads to different definitions of feedbacks and ultimately differing quantitative assessment of feedback strengths.

### Limitations of the linear approach

The standard radiative feedback framework assumes that the response of the system can be expressed as a linear function of the surface temperature. It is a very useful approximation but some processes cannot be expressed in terms of functions of single variables and the radiative feedback framework has to be adjusted to capture changes in the system not directly related to surface temperature^91, 92^. Moreover, assuming linearity in feedbacks fails in many cases, as can be expected for a system as complex as the Earth’s climate. For example, the magnitude of the climate feedback parameter *λ* generally decreases with time in climate models after a rise in atmospheric CO_2_ concentration, corresponding to increasing climate sensitivity as equilibrium is approached^32, 76, 92^. *λ* may also depend on the magnitude of the perturbation^87, 93–95^.

The non-linearity of the feedbacks can be described in different ways. A simple definition will be used here: the feedback is non-linear if the feedback factor *γ* is not constant. Non-linearities can be caused by several processes. The strength of the feedback can be a function of the state of the system. This state dependence can often be expressed as a time dependence when the state changes with time. Furthermore, the different processes controlling the response to a perturbation may have different time scales. Their relative contribution to local and global scale feedbacks may thus evolve leading to spatially or temporally non-constant feedback factors^76, 92, 96^.

**Fig. 2:**
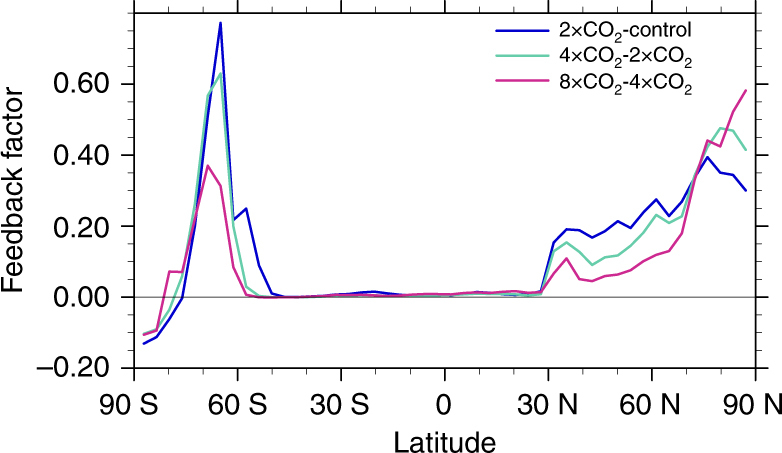
Nonlinearity in the surface albedo feedback factor for three consecutive doublings of CO_2_. The feedback factor, defined as the ratio of the magnitude of the albedo feedback on the Planck feedback, is calculated using the radiative kernel technique^85^ and zonal averages are plotted for three consecutive doublings of CO_2_ concentrations in CCSM3. The global average feedback factor decreases^87^ from 0.097 for 2xCO_2_–CNTL to 0.053 for 8xCO_2_–4xCO_2_

In polar regions, the presence of different phases of water implies that many feedback parameters display a particularly strong dependence on the state of the system near the freezing point and are thus highly non-linear. For instance, phase changes play an important role in polar clouds leading to non-linearities in the cloud feedback^32, 34, 39, 40^. Furthermore, feedbacks related to the cryosphere generally depend on the surface area covered by snow or ice. As temperatures rise, this area decreases and the feedback strength is reduced. This is illustrated^87^ in Fig. 2 for the surface albedo feedback in response to three consecutive doublings of CO_2_ in the Community Climate System Model version 3 (CCSM3). At many latitudes, the value of the feedback factor is smaller for the third doubling (8 × CO_2_–4 × CO_2_) than it is for the first (2 × CO_2_–CNTL). Between 50°S and 60°S the feedback approaches zero for the third doubling, since the Southern Ocean is already ice-free at these latitudes in the 4xCO_2_ climate, and no further melting can occur. On the other hand, the value of the feedback factor increases at northern high latitudes (75°N–90°N), as the sea ice edge retreats within the central Arctic at high warming.

### Non-radiative feedbacks

The traditional radiative feedback framework has been extended to additional processes that influence the energy balance of the Earth, offering an effective way to evaluate and compare the strength of the different feedbacks^55, 76, 89^. This approach has also been successful for some biogeochemical and biogeophysical feedbacks^66^.

In contrast, the evaluation of key non-radiative polar feedbacks is generally inconsistent among the different feedbacks and even among different studies of the same feedback. For the ice growth–thickness feedback, in analogy with the radiative feedback framework, the thickness sensitivity parameter is defined as the ratio of the sea ice thickness change to the perturbative forcing^49^, but this definition has not been widely used so far. The effectiveness of the ice production-entrainment feedback can be measured^50, 51^ as the ratio between the melting immediately caused by the entrainment of warmer water in the surface layer to the initial ice growth. The ice production-ocean heat storage feedback has been estimated by the ratio between the heat losses associated with sea-ice volume changes to the heat storage below the surface level^52, 53^. Both quantities can be evaluated directly from observations or model results. (Supplementary Note 1). Those definitions appear justified taken alone but the diversity of definitions and methods to quantify those feedbacks complicates their systematic evaluation and the comparison of the role of the different processes in observed changes. A common framework would thus be very helpful.

## Implications of correctly quantifying feedbacks

The analysis and quantification of feedbacks have many potential applications. This is illustrated in this section by explaining how this can be used to understand the higher temperature changes expected in the Arctic compared to other regions, to perform a process-oriented evaluation of model behavior, and to reduce the uncertainty in projections.

### Polar amplification

**Fig. 3:**
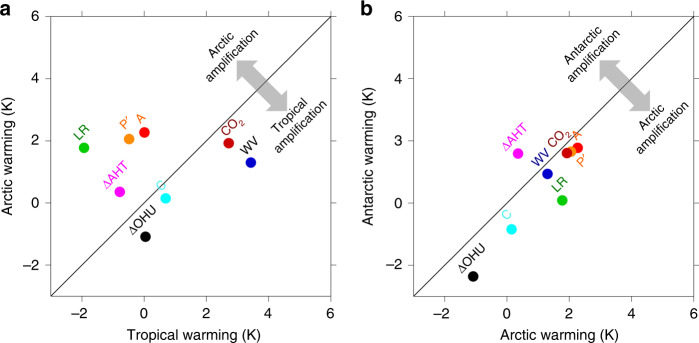
Contributions of each feedback and atmospheric forcing to polar amplification. **a** Arctic (60–90N) relative to tropics (30S–30N), and **b** Antarctic (60–90S) relative to Arctic (60–90N) at year 100 of abrupt CO_2_ quadrupling in climate models involved in the fifth phase of the Coupled Model Intercomparison Project (CMIP5). The feedbacks shown are the lapse rate (LR), surface albedo (A), water vapor (WV), cloud (C), and latitudinal variation in the Planck response (P’, local difference from its global-mean value *λ*_0_); the additional energetic contributions shown are the CO_2_ forcing (CO_2_), atmospheric heat transport convergence (ΔAHT) and ocean heat uptake (ΔOHU) (see method section). The feedbacks are expressed as warming contributions to the total temperature change

Overall, climate feedbacks are less stabilizing (i.e., feedback parameters are less negative or more positive) in polar regions than in the tropics. This explains the larger temperature changes experienced in polar regions in response to a perturbation (Fig. 3), a phenomenon referred to as polar amplification^19, 20, 23, 97^. For the climate changes projected for the 21st century, polar amplification is much stronger in the Arctic than in the Antarctic. In the Arctic, the large amplification mostly results from (1) a relatively large and positive lapse rate feedback, due to a different vertical distribution of the temperature change compared to the tropics; (2) a relatively weak negative Planck response, due to smaller blackbody emissions per unit warming at lower temperatures (Stefan–Boltzmann law); and (3) a large positive surface albedo feedback, due to the loss of high albedo snow and ice-covered surfaces, as well as a contribution from atmospheric heat transport (Fig. 3a). In the Antarctic, both the weak Planck response and the positive surface albedo feedback induce polar amplification. Warming is damped relative to the Arctic due to a less positive lapse rate feedback, more negative cloud feedback, and strong ocean heat uptake in the Southern Ocean under transient warming (Fig. 3b).

Note that if feedbacks are defined using global mean rather than local surface temperature change, the Planck feedback appears strongly negative in the Arctic because the local temperature change exceeds the local mean. Additionally, the polar amplification has a large seasonal cycle, displaying over the Arctic a minimum in summer and a maximum in fall/winter. In summer, the influence of the large positive surface albedo feedback is compensated by a strong oceanic heat uptake and negative cloud feedbacks while in fall/winter the heat released by the ocean, the contribution of lapse rate feedback and cloud feedbacks induce a large warming^55, 98^.

### Origin of model biases

In polar regions, many studies have identified model strengths and weaknesses in reproducing observations^1, 8, 10, 99, 100^. Yet, perhaps the most important challenge is identifying the climate system processes that must be represented in order to consider a model realistic enough for mechanistic studies and projections. More generally, simple comparisons between model results and observations do not allow estimating the origin of model biases or their impact. Process-based model evaluation offers the possibility of exploring the causes of discrepancies more deeply, identifying the links between various variables and ultimately suggesting model improvements^8, 46, 101–103^.

**Fig. 4:**
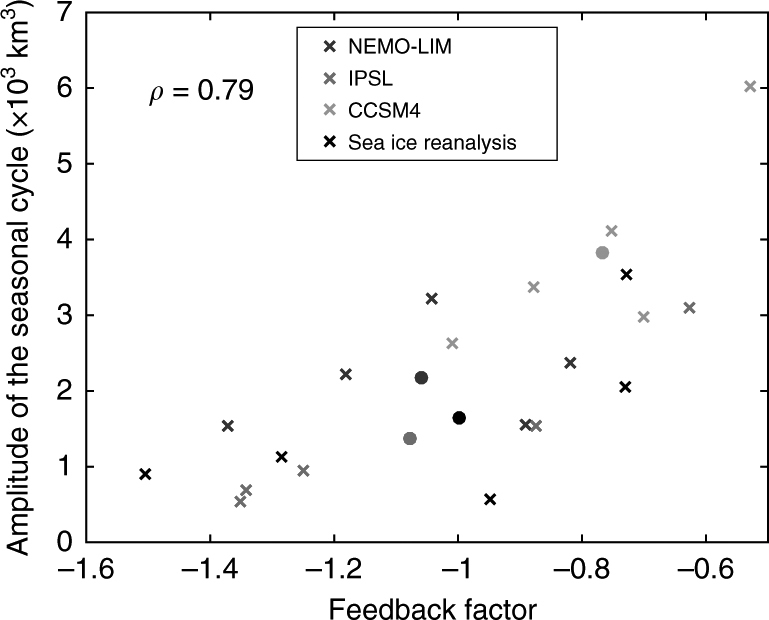
Amplitude of the sea ice volume seasonal cycle versus the ice production-entrainment feedback factor. The ice production-entrainment feedback factor *γ*_*θ*_ is defined as the ratio of the melting due to warm water entrainment to the initial ice formation^50^. The values are estimated over 1990–2005 for a standardized perturbation corresponding to an increase of 10 cm of sea ice. For both observational and model datasets, the evaluation of *γ*_*θ*_ is performed on the basis of temperature and salinity profiles in the Southern Ocean, averaged over January–February for the period 1990–2005. Values are represented by five crosses corresponding to five sectors of the Southern Ocean^111^. Results for NEMO-LIM^112^, CCSM4^113^, and IPSL^114^ models are in colors. Estimates are given in black based on oceanic observations^115^ and the sea ice volume derived from a reanalysis^116^. For all datasets, the plain circles correspond to the average of *γ*_*θ*_ and of the amplitude of the ice volume seasonal cycle over all sectors, and *ρ* is the correlation coefficient between these two quantities

To illustrate this point, we compare the ice production–entrainment feedback in three existing simulations for all the sectors of the Southern Ocean with estimates derived from observations and a reanalysis (Fig. 4). A clear link is found between the value of the feedback factor and the amplitude of the seasonal cycle of ice volume: since the ice production–entrainment feedback is negative, it tends to damp the seasonal cycle; i.e., a stronger feedback corresponds to a weaker seasonal cycle. The spread across simulations and Antarctic sea ice regions in Fig. 4 stresses the large sensitivity of the feedback to the ocean properties. As many climate models suffer from large biases in their representation of the vertical structure of the Southern Ocean, they are unlikely to predict this feedback accurately. For instance, the overestimation of the amplitude of the seasonal cycle of sea ice volume in the model CCSM4 is likely related to a too weak negative feedback and improvements in the representation of ocean properties, in particular of temperature and salinity below the surface layer, would reduce this bias.

### Uncertainties in model projections

One justification of the development of the radiative feedback framework is to determine the processes that can explain the range of model projections for a specific scenario of future changes in radiative forcing. As expected, the models displaying the largest surface temperature changes are the ones for which the radiative feedbacks have the largest (most positive) values. The same approach can be applied to the non-radiative feedbacks investigated here as they are related to the magnitude of the response to any type of perturbation.

Although it is better if a model is able to reproduce the observations with a bias that is as small as possible, it is not clear for many variables, such as the global mean temperature, that the response to a perturbation is a function of this bias^104^. In other words, there is no a priori reason to believe that a model which reproduces the present global mean temperature well will provide more reasonable projections of future climate than a model that has larger biases. Indeed, a model may have a global mean temperature close to observations due to compensations between many factors that may not necessarily balance in a projected climate^105^.

The situation is distinctive for polar regions, where the feedbacks are strongly non-linear and thus state dependent. This provides an instructive way to interpret the range of model responses as a function of the value of some variables for present-day conditions^106, 107^. Furthermore, it has been argued that a model displaying a more realistic mean state in polar regions will also have a better representation of key processes and thus will provide a more likely estimate of future climate changes than a model with larger biases. This has then been used to justify the selection of models based on their mean state^108, 109^. This idea appears useful in principle but is hard to generalize and is subject to criticism. In particular, it is not always clear to determine how to evaluate models, which variable should be used to select models, and if the currently available model sample is adequate to apply a meaningful selection. This has led to strong debate in the community about the justification of this approach, which may artificially reduce the uncertainty range by discarding model results that are as likely as the others^110^. We propose to use feedback quantification more extensively to evaluate model behavior, and foresee that this can contribute to more robust estimates of the likelihood of projections.

## A simple and consistent approach for non-radiative feedbacks

The review above illustrates that many definitions and evaluation methods have been proposed for the various radiative and non-radiative feedbacks. Nevertheless, all the feedbacks can be described and quantified using a simple and consistent framework, based on the definition of a feedback factor *γ*.

For radiative feedbacks, the feedback factor *γ*_*i*_ is the ratio of a particular feedback parameter to minus the Planck feedback parameter. An analogous expression can be written for any other feedback. When only one feedback is operating (see the methods for the case of multiple feedbacks), the feedback factor *γ* can be quantified as the ratio between the additional changes specifically due to the feedback and the response of the full system including all the feedbacks (Total response). This additional change (Total response − Reference response) is itself computed as the difference between the response of the full system and the one of a reference system in which the feedback under consideration does not operate (Reference response):

1 $$\upgamma = \frac{\mathrm{Total{\kern 2pt} response - Reference{\kern 2pt} response }}{\mathrm{Total{\kern 2pt} response}}$$

The methodology requires explicitly identifying (1) a perturbation or a class of perturbations, (2) a response variable involved in the feedback loop, (3) the full system with all processes operating and its response to the perturbation, and (4) the reference system with the process of interest not operating and the reference system response to the perturbation. While the framework is general, a clear definition of this system is required, as the value of the feedback factor depends on the way the reference system is chosen^12^. Let’s examine radiative feedbacks as one example (see the methods): (1) the perturbation is the radiative forcing *F*, (2) the response variable is *T*_*s*_, (3) the full system includes one or more radiative feedbacks plus the Planck response referenced to *T*_*s*_, and (4) the reference system is the Planck response only.

As it is based on the same principles, analysis of non-radiative processes using this feedback factor retains the main advantages of the radiative feedback framework. First, each feedback can be associated with a well-defined conceptual model describing the mechanisms and interactions involved. This is essential in order to allow each feedback to be firmly rooted in a process-based analysis that is straightforward to apply and understand. Secondly, it is possible to evaluate the magnitude of the feedbacks using a dimensionless factor, ideally both in models and observations. This is required to assess the contributions of the different feedbacks to the total response and to compare the role of each feedback in various Earth System models to determine which is responsible for their distinctive sensitivities.

As in the example below, the feedback factor can in some cases be evaluated using observations or model outputs only, but it may also require specific additional calculations. This is illustrated using a simple model in Supplementary Note 2 for the ice growth-ice thickness feedback. In this case, potential compensations can occur between feedbacks and the interpretation of the estimates that are obtained must then take into those synergies^65, 89^.

### An example of the approach for the ice production-ocean entrainment feedback

We illustrate the methodology with the ice production–entrainment feedback^50^. For this negative feedback, (1) the perturbation is a given amount of ice production, (2) the reference variable is ice thickness, (3) the full system is the sea ice plus ocean column with the entrainment process, and (4) the reference system is the sea ice plus ocean column but without entrainment. The intensity of this feedback can then be evaluated using the ratio

2 $$\upgamma _\theta = {\textstyle{{{\mathrm{Total}}{\kern 2pt} {\mathrm{ice}}{\kern 2pt} {\mathrm{thickness}}{\kern 2pt} {\mathrm{changes}} - {\mathrm{Ice}}{\kern 2pt} {\mathrm{thickness}}{\kern 2pt} {\mathrm{changes}}{\kern 2pt} {\mathrm{without}}{\kern 2pt} {\mathrm{entrainment}}} \over {{\mathrm{Total}}{\kern 2pt} {\mathrm{ice}}{\kern 2pt} {\mathrm{thickness}}{\kern 2pt} {\mathrm{changes}}}}}$$

Despite a different form, this expression is strictly equivalent to the original formulation proposed in ref. ^50^ and used for Fig. 4 (see Supplementary Note 1 for the demonstration).

**Fig. 5:**
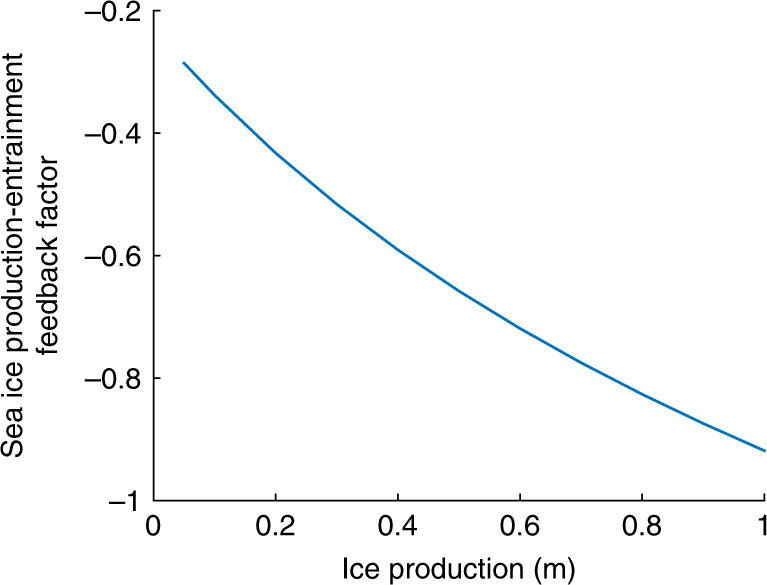
Evolution of the ice production-entrainment feedback factor as a function of ice production. For each value of ice production, the entrainment is computed from the January–February 1990–2005 mean temperature and salinity profiles^115^ assuming a mixed layer deepening restoring the static stability of the water column after the brine release. It is shown here for a Weddell Sea location typically covered by ice in winter (near 30°W, 65°S). The strength of the pycnocline is thus evaluated in summer but it must be measured below the layer close to the surface that is warmed above freezing point temperature if sea ice completely melts, as the heat in this layer is removed quickly in fall when the temperature drops and is not involved in the ice production-ocean entrainment feedback

As the mixed layer deepens, it entrains water with increasing temperature (since temperature increases with depth) and the heat input grows. Consequently, the absolute value of the feedback factor *γ*_*θ*_ increases with ice formation (meaning that its value decreases, since it is a negative feedback) until the end of winter (Fig. 5). This non-linear behavior can be illustrated using a simple analytical model as shown in Supplementary Note 3 and Supplementary Fig. 3.

In practice, it is usually not possible to completely quantify all the dependencies of a non-linear, spatially variable feedback factor. This is why it has been suggested to select a prescribed perturbation representative of the condition of interest, similarly to the classical analysis of radiative forcing in terms of doublings of atmospheric CO_2_ concentrations (Fig. 2). For the ice production-ocean entrainment feedback, we propose to evaluate *γ*_*θ*_ by considering the response to a standardized perturbation corresponding to an increase in sea ice thickness of 10 cm (Fig. 4). The number of observed profiles being much higher in summer, the feedback parameter is evaluated from data collected during this season. It is clear that the corresponding values of the feedback factors are not universally valid but they provide a standard benchmark for comparisons and analyses.

## Concluding remarks

This Perspective underlines the critical role of feedbacks in the dynamics of polar climate and the need to quantify them precisely. Feedback quantification provides a powerful tool to understand the interactions between the components of the system, to analyze model biases and to determine the origin of the differences within a set of model predictions. We have focused on some physical processes affecting the atmosphere, sea ice, ice sheets, land surfaces and ocean in polar regions. Yet, the discussion can be extended to feedbacks including biogeochemical processes.

Quantification of feedback strength is not simple as many polar feedbacks are strongly non-linear. Indeed, feedback magnitude depends on the location, the season and is a function of the climate state. We have provided here values in specific conditions for some of the feedbacks investigated. However, one single number or a range, as could be included in a table for instance, is not sufficient to fully characterize a feedback and its variations.

While the quantitative estimation of feedbacks follows well-established methodologies for radiative feedbacks, this is not the case for many other feedbacks. Nevertheless, the traditional radiative feedback analysis can be extended to define a feedback factor that can be used as a standard measure of most polar feedbacks. This feedback factor is estimated as the relative contribution of the feedback to the total change of the system in response to a perturbation. It has several advantages: (1) it is compatible with the radiative feedback framework which has proven useful over the past three decades, (2) it is based on a clear physical interpretation of key physical processes, (3) the framework is simple to articulate, and (4) an unambiguous quantification can be obtained allowing an objective evaluation of the processes that control the response to a perturbation.

Using a feedback factor provides the clear theoretical advantages of being consistent with the feedback theory, general and easy to interpret. Nevertheless, based on data availability or on the specific goal of a study, other parameters will continue to be used to diagnose the magnitude of some feedbacks. This may of course be perfectly justified. However, the limitations and merit of such an approach compared to a more general view need to be discussed and taken into account when interpreting the results.

Even though the feedbacks discussed here are well-known, some are often only used to provide a qualitative interpretation or a narrative framework to explain the changes occurring in polar regions. In particular, the quantitative evaluation of non-radiative feedbacks in models is rare, with only few and, in some cases, no publications on this topic for some feedbacks. This Perspective motivates the use of more systematic approaches to analyze past and upcoming model results. In particular, the framework presented here allows tracing the origin of model diversity back to physical considerations. Identifying the feedbacks that are critical for correctly simulating the mean state and variability of polar climate will ultimately promote the development of targeted observational campaigns, by means of which models will be evaluated. Such campaigns already exist or are underway: the Year of Polar Prediction (YOPP) or the Multidisciplinary drifting Observatory for the Study of Arctic Climate (MOSAiC) are two examples. In summary, advancing evaluation of feedbacks will require dedicated model experiments and careful analyses, complemented with the collection of dedicated observations that can constrain model feedbacks. Some of those elements are clearly challenging, but this will be strongly beneficial for our understanding of polar climate dynamics and of the future changes expected at high latitudes.

## Methods

### Radiative feedbacks expressed as feedback factors

The global mean radiative balance at the TOA in response to a radiative forcing *F* (in W m^−2^) at equilibrium can be written as

3 $$F + \left( {\lambda _{_0} + \mathop {\sum}\limits_i {\lambda _i} } \right){\mathrm{\Delta }}T_{\mathrm{S}} = 0$$

where Δ*T*_S_ (in K) is the surface temperature change, *λ*_0_ (~−3.2 W m^−2^ K^−1^) the Planck response and the *λ*_*i*_ (in W m^−2^ K^−1^) correspond to the radiative feedback parameters related to the response of surface albedo, clouds, water vapor and vertical temperature gradient (lapse rate feedback).

In the absence of these feedbacks, the equilibrium surface temperature change in response to a doubling of CO_2_ would be governed by only the Planck response, given by${\mathrm{\Delta }}T_{\mathrm{0}} = - F{\mathrm{/}}\lambda _{_0}$and equal to approximately^12^ 1.2 K. This temperature change is amplified or damped by individual feedbacks, depending on whether they contribute to a positive or negative additional radiative perturbation to the TOA radiative balance in response to warming. The overall equilibrium warming resulting from a CO_2_ doubling is thus greater than Δ*T*_0_, likely^12–14^ between about 2 and 4.5 K.

The primary advantage of the feedback framework is that it allows a process-based analysis of the adjustment of the system to a radiative perturbation and a quantification of the importance of each process. Traditionally, the magnitude of each feedback is compared to that of the Planck response, giving dimensionless feedback factors *γ*_*i*_ = *λ*_*i*_/−*λ*_0_. In turn, the surface temperature change in response to forcing can be cast in terms of the feedback factors as

4 $${\mathrm{\Delta }}T_{\mathrm{s}} = {\mathrm{\Delta }}T_{\mathrm{0}}{\mathrm{/}}\left( {1 - \mathop {\sum}\limits_i {\gamma _i} } \right)$$

The sum of all the feedback factors $\gamma _g = \mathop {\sum}\limits_i {\gamma _i} = \mathop {\sum}\limits_i {\frac{{\lambda _i}}{{ - \lambda _0}}}$ can also be calculated as

5 $$\gamma _g = \frac{{{\mathrm{\Delta }}T_{\mathrm{s}} - {\mathrm{\Delta }}T_0}}{{{\mathrm{\Delta }}T_{\mathrm{s}}}}$$

This sum can thus be interpreted as the additional warming due to the feedbacks divided by the total temperature change.

### Radiative feedbacks expressed as warming contributions

The formalism can be extended to assess the relative contributions of individual feedbacks to local surface warming by use of the local energy budget equation:

6 $$F + \left( {\uplambda _{_0} + \mathop {\sum}\limits_{\rm i} {\uplambda _{\rm i}} } \right){\mathrm{\Delta }}T_{\mathrm{S}} + {\mathrm{\Delta OHU}} + {\mathrm{\Delta AHT}} = 0$$

where each variable is a function of latitude, ΔOHU is the change in ocean heat uptake (positive into atmospheric column) and ΔAHT represents the change in atmospheric heat flux convergence (positive into atmospheric column). Following previous studies^18, 19, 72^, we define the warming contribution as the energetic contribution (in W m^−2^) associated with a particular feedback (*λ*_*i*_Δ*T*_s_) or atmospheric forcing (*F*, ΔOHU or ΔAHT) divided by the magnitude of the global-mean Planck response $- \overline {\lambda _0}$:

7 $${\mathrm{\Delta }}T_{\mathrm{s}} = - F/\overline {\uplambda _0} - \uplambda _0^\prime {\mathrm{\Delta }}T_{\mathrm{s}}{\mathrm{/}}\overline {\uplambda _0} - \mathop {\sum}\limits_i {\uplambda _i\Delta T_{\mathrm{s}}} {\mathrm{/}}\overline {\uplambda _0} - {\mathrm{\Delta OHU}}{\mathrm{/}}\overline {\uplambda _0} - {\mathrm{\Delta AHT}}{\mathrm{/}}\overline {\uplambda _0}$$

where the terms on the right-hand side each represent an individual warming contribution and together sum to the total surface warming Δ*T*_s_ (with small residual ignored here); $\lambda _0^\prime = \lambda _0 - \overline {\lambda _0}$ represents the deviation of the local Planck response from its global-mean value. Here we use local feedbacks *λ* diagnosed using radiative kernels^85^ averaged over years 85–115 of abrupt CO_2_ quadrupling simulations of 13 models^72^; ΔOHU is diagnosed as the anomalous net surface heat flux and ΔAHT as the difference between ΔOHU and net TOA radiation flux anomalies. Figure 3 shows the calculated warming contributions of feedbacks and forcings in polar and tropical regions.

### Feedback factor and the feedback gain when several feedbacks are operating

When several feedbacks are operating, their contribution to the changes in the radiative balance is additive. Using the same notations as above, the radiative balance at equilibrium is

8 $$F + \left( {\lambda _{_0} + \mathop {\sum}\limits_i {\lambda _i} } \right){\mathrm{\Delta }}T_{\mathrm{S}} = 0$$

leading when dividing by *λ*_0_ to

9 $$\frac{F}{{\lambda _{_0}}} + \left( {1 + \mathop {\sum}\limits_i {\frac{{\lambda _i}}{{\lambda _{_0}}}} } \right){\mathrm{\Delta }}T_{\mathrm{S}} = 0$$

and

10 $$- {\mathrm{\Delta }}T_0 + \left( {1 - \mathop {\sum}\limits_i {\gamma _i} } \right){\mathrm{\Delta }}T_{\mathrm{S}} = 0$$

The contribution of the feedback factors is thus also additive.

This is not the case for feedback gain *G*

11 $$G = \frac{{{\mathrm{\Delta }}T_{\mathrm{s}}}}{{{\mathrm{\Delta }}T_0}} = \frac{1}{{1 - \mathop {\sum}\limits_i {\gamma _i} }} = \frac{1}{{1 - \gamma _g}}$$

As the various feedbacks are interacting to get the full response, the gain when two feedbacks are active is not the sum of the gains when the feedbacks are acting separately.

When only one feedback is acting or if only the sum of all feedbacks is considered, *γ*_*g*_ can be simply evaluated by

12 $$\gamma _g = \frac{{{\mathrm{\Delta }}T_{\mathrm{s}} - {\mathrm{\Delta }}T_0}}{{{\mathrm{\Delta }}T_{\mathrm{s}}}}$$

Several techniques are available^82, 85^ to estimate the individual *γ*_*i*_. They generally require to perform specific analyses to extract the contribution of a particular feedback. One example is to perform an experiment when only the investigated feedback is operating and comparing the changes Δ*T*_*si*_ in this experiment to the one of the reference system Δ*T*_0_.

13 $$\gamma _i = \frac{{{\mathrm{\Delta }}T_{{\mathrm{s}}i} - {\mathrm{\Delta }}T_{\mathrm{0}}}}{{{\mathrm{\Delta }}T_{{\mathrm{s}}i}}}.$$

This solution is not the one traditionally used for radiative feedbacks but the approach can be generalized to any feedback where this alternative may be a practical solution for feedback evaluation.

## Electronic supplementary material

Supplementary Information

## References

[1] Stroeve JC (2012). The Arctic’s rapidly shrinking sea ice cover: a research synthesis. Clim. Change.

[2] Stammerjohn S, Massom R, Rind D, Martinson D (2012). Regions of rapid sea ice change: an inter-hemispheric seasonal comparison. Geophys. Res. Lett..

[3] Döscher R, Vihma T, Maksimovich E (2014). Recent advances in understanding the Arctic climate system state and change from a sea ice perspective: a review. Atmos. Chem. Phys..

[4] Armour KC, Marshall J, Scott J, Donohoe A, Newsom ER (2016). Southern Ocean warming delayed by circumpolar upwelling and equatorward transport. Nat. Geos..

[5] Jones JM (2016). Assessing recent trends in high-latitude Southern Hemisphere surface climate. Nat. Clim. Change.

[6] Hobbs WR (2016). A review of recent changes in Southern Ocean sea ice, their drivers and forcings. Glob. Planet. Change.

[7] Swart NC, Fyfe JC, Hawkins E, Kay JE, Jahn A (2015). Influence of internal variability on Arctic sea-ice trends. Nat. Clim. Change.

[8] Notz, D. How well must climate models agree with observations? *Phil.Trans. R. Soc. A* **373**, 20140164. 10.1098/rsta.2014.0164 (2015). 10.1098/rsta.2014.0164 PMC4607702 26347535

[9] Zunz V, Goosse H, Massonnet F (2013). How does internal variability influence the ability of CMIP5 models to reproduce the recent trend in Southern Ocean sea ice extent?. Cryosphere.

[10] Flato, G. et al. *Evaluation of Climate Models. Climate Change: The Physical Science Basis. Contribution of Working Group I to the Fifth Assessment Report of the Intergovernmental Panel on Climate Change* (eds Stocker,T.F., Qin, D., Plattner, G.-K., Tignor, M., Allen, S. K., Boschung, J., Nauels, A., Xia, Y., Bex, V., & Midgley, P.M.) (Cambridge United Kingdom and New York, USA, 2013).

[11] Åström, K. J. & Murray, R. M. *Feedback systems: an introduction for scientists and engineers*. (Princeton, New Jersey, USA, 2010) (ISBN 9781400828739).

[12] Roe GH (2009). Feedbacks, timescales and seeing red. Annu. Rev. Earth. Planet. Sci..

[13] Hansen, J. E. et al. Climate sensitivity: analysis of feedback mechanisms. *Climate processes and climate sensitivity* (eds Hansen, J. E. & Takahashi, T.) 130–163 (American Geophysical Union, Washington, DC, USA, 1984).

[14] Bony S (2006). How well do we understand and evaluate climate change feedback processes?. J. Clim..

[15] Wallace, J. M. & Hobbs, P. V. Atmospheric science: an introductory survey 2nd edn. *International Geophysics Series* **92**, 484 (Academic press, Burlington, MA, USA and San Diego, CA, USA, 2006).

[16] Armour KC, Bitz CM, Roe GH (2013). Time-varying climate sensitivity from regional feedbacks. J. Clim..

[17] Ramanathan V (1977). Interactions between ice-albedo, lapse-rate and cloud-top feedbacks: an analysis of the nonlinear response of a GCM climate model. J. Atmos. Sci..

[18] Crook JA, Forster PM, Stuber N (2011). Spatial patterns of modeled climate feedback and contributions to temperature response and polar amplification. J. Clim..

[19] Pithan F, Mauritsen T (2014). Arctic amplification dominated by temperature feedbacks in contemporary climate models. Nat. Geosci..

[20] Manabe S, Wetherald R (1975). The effects of doubling the CO_2_ concentration on the climate of a general circulation model. J. Atmos. Sci..

[21] Dessler AE, Zhang Z, Yang P (2008). Water–vapor climate feedback inferred from climate fluctuations, 2003–2008. Geophys. Res. Lett..

[22] Gordon ND, Jonko AK, Forster PM, Shell KM (2013). An observationally based constraint on the water–vapor feedback. J. Geophys. Res. Atmos..

[23] Taylor PC (2013). A decomposition of feedback contributions to polar warming amplification. J. Clim..

[24] Graversen RG, Wang M (2009). Polar amplification in a coupled climate model with locked albedo. Clim. Dyn..

[25] Budyko M (1969). The effect of solar radiation variations on the climate of the Earth. Tellus.

[26] Sellers P (1969). A global climate model based on the energy balance of the earth-atmosphere system. J. Appl. Meteor..

[27] Hall A (2004). The role of surface albedo feedback in climate. J. Clim..

[28] Winton M (2006). Surface albedo feedback estimates for the AR4 climate models. J. Clim..

[29] Perovich DK (2003). Thin and thinner: sea ice mass balance measurements during SHEBA. J. Geophys. Res. Oceans.

[30] Sturm, M. & Massom, R. A. Snow in the sea ice system: friend or foe? In *Sea Ice* (ed. Thomas, D. N.) 65–109 (Wiley-Blackwell, Oxford, United Kingdom, 2017).

[31] Wetherald RT, Manabe S (1988). Cloud feedback processes in a general circulation model. J. Atmos. Sci..

[32] Mitchell JFB, Senior CA, Ingram WJ (1989). On CO_2_ and climate: a missing cloud feedback?. Nature.

[33] Vial J, Dufresne JL, Bony S (2013). On the interpretation of inter-model spread in CMIP5 climate sensitivity estimates. Clim. Dyn..

[34] Zelinka MD, Klein SA, Hartmann DL (2012). Computing and partitioning cloud feedbacks using cloud property histograms. Part II: attribution to changes in cloud amount, altitude, and optical depth. J. Clim..

[35] Andrews T, Gregory JM, Webb MJ, Taylor KE (2012). Forcing, feedbacks and climate sensitivity in CMIP5 coupled atmosphere–ocean climate modelsPlease check whether the author names in reference 35 are correct, and confirm.OK. Geophys. Res. Lett..

[36] Schweiger AL, Lindsay RW, Vavrus S, Francis JA (2008). Relationships between Arctic sea ice and clouds during Autumn. J. Clim..

[37] Morrison AL, Kay JE, Chepfer H, Guzman R, Yettella V (2018). Isolating the liquid cloud response to recent Arctic sea ice loss using spaceborne lidar observations. J. Geophys. Res. Atmos..

[38] Kay JE (2016). Recent advances in Arctic cloud and climate research. Curr. Clim. Change Rep..

[39] Boisvert LN, Wu DL, Shie CL (2015). Increasing evaporation amounts seen in the Arctic between 2003 and 2013 from AIRS data. J. Geophys. Res. Atmos..

[40] Bodas-Salcedo A, Andrews T, Karmalkar AV, Ringer MA (2016). Cloud liquid water path and radiative feedbacks over the Southern Ocean. Geophys. Res. Lett..

[41] Wall CJ, Kohyama T, Hartmann DL (2017). Low-cloud, boundary layer, and sea ice interactions over the Southern Ocean during winter. J. Clim..

[42] Terai CR, Zelinka M, Klein SA (2016). Constraining the low-cloud optical depth feedback at middle and high latitudes using satellite observations. J. Geophys. Res. Atmos..

[43] Ceppi P, McCoy DT, Hartmann DL (2016). Observational evidence for a negative shortwave cloud feedback in mid to high latitudes. Geophys. Res. Lett..

[44] Bodas-Salcedo A (2014). Origins of the solar radiation biases over the Southern Ocean in CFMIP2 models. J. Clim..

[45] Tan I, Storelvmo T, Zelinka MD (2016). Observational constraints on mixed-phase clouds imply higher climate sensitivity. Science.

[46] Kay JE (2016). Evaluating and improving cloud phase in the Community Atmosphere Model version 5 using spaceborne lidar observations. J. Geophys. Res. Atmos..

[47] Holland MM, Bitz CM, Tremblay B (2006). Future abrupt reductions in the summer Arctic sea ice. Geophys. Res. Lett..

[48] Maykut, G. A. The surface heat and mass balance. *The Geophysics of Sea Ice* (ed. Untersteiner, N.) 395–464 (Plenum Press, 1986).

[49] Bitz CM, Roe GH (2004). A mechanism for the high rate of sea ice thinning in the Arctic Ocean. J. Clim..

[50] Martinson DG (1990). Evolution of the Southern Ocean winter mixed layer and sea ice-open ocean deep-water formation and ventilation. J. Geophys. Res. Oceans.

[51] Martinson, D. G. & Iannuzzi, R. A. Antarctic ocean–ice interaction: implications from ocean bulk property distributions in the Weddell Gyre. *Antarctic sea ice: physical processes, interactions and variability*, (ed. Jeffries, M.) 243–271 (American Geophysical Union, Washington, DC, USA, 1998).

[52] Goosse H, Zunz V (2014). Decadal trends in the Antarctic sea ice extent ultimately controlled by ice–ocean feedback. Cryosphere.

[53] Lecomte O (2017). Vertical ocean heat redistribution sustaining sea-ice concentration trends in the Ross Sea. Nat. Comm..

[54] Seneviratne SI (2010). Investigating soil moisture–climate interactions in a changing climate: a review. Earth-Sci. Rev..

[55] Laîné A, Yoshimori M, Abe-Ouchi A (2016). Surface Arctic amplification factors in CMIP5 models: land and oceanic surfaces and seasonality. J. Clim..

[56] Edwards TL (2014). Effect of uncertainty in surface mass balance–elevation feedback on projections of the future sea level contribution of the Greenland ice sheet. Cryosphere.

[57] Edwards TL (2014). Probabilistic parameterization of the surface mass balance–elevation feedback in regional climate model simulations of the Greenland ice sheet. Cryosphere.

[58] Schoof C (2007). Ice sheet grounding line dynamics: steady states, stability, and hysteresis. J. Geophys. Res..

[59] Docquier D, Perichon L, Pattyn F (2011). Representing grounding line dynamics in numerical ice sheet models: recent advances and outlook. Surv. Geophys..

[60] Rignot E, Mouginot J, Morlighem M, Seroussi H, Scheuchl B (2014). Widespread, rapid grounding line retreat of Pine Island, Thwaites, Smith, and Kohler glaciers, West Antarctica, from 1992 to 2011. Geophys. Res. Lett..

[61] Wise MG, Dowdeswell JA, Jakobsson M, Larter RD (2017). Evidence of marine ice-cliff instability in Pine Island Bay from iceberg-keel plough marks. Nature.

[62] Zwally HJ (2002). Surface melt-induced acceleration of Greenland ice-sheet flow. Science.

[63] Swingedouw D (2008). Antarctic ice-sheet melting provides negative feedbacks on future climate warming. Geophys. Res. Lett..

[64] Bintanja R, van Oldenborgh GJ, Drijfhout SS, Wouters B, Katsman CA (2013). Important role for ocean warming and increased ice-shelf melt in Antarctic sea-ice expansion. Nat. Geos..

[65] Otto J, Raddatz T, Claussen M, Brovkin V, Gayler V (2009). Separation of atmosphere-ocean-vegetation feedbacks and synergies for mid-Holocene climate. Geophys. Res. Lett..

[66] Gregory JM, Jones CD, Cadule P, Friedlingstein P (2009). Quantifying carbon cycle feedbacks. J. Clim..

[67] Arneth A (2010). Terrestrial biogeochemical feedbacks in the climate system. Nat. Geosci..

[68] Schuur EAG (2015). Climate change and the permafrost carbon feedback. Nature.

[69] Pabi S, van Dijken GL, Arrigo KR (2008). Primary production in the Arctic Ocean, 1998–2006. J. Geophys. Res..

[70] Lengaigne M (2009). Bio-physical feedbacks in the Arctic Ocean using an Earth system model. Geophys. Res. Lett..

[71] Alexeev VA, Jackson CH (2013). Polar amplification: is atmospheric heat transport important?. Clim. Dyn..

[72] Feldl N, Bordoni S, Merlis TM (2017). Coupled high-latitude climate feedbacks and their impact on atmospheric heat transport. J. Clim..

[73] Kay JE (2012). The influence of local feedbacks and northward heat transport on the equilibrium Arctic climate response to increased greenhouse gas forcing in coupled climate models. J. Clim..

[74] Roe GH, Feldl N, Armour KC, Hwang YT, Frierson DMW (2015). The remote impacts of climate feedbacks on regional climate predictability. Nat. Geosci..

[75] Cai M, Lu J (2007). Dynamical greenhouse-plus feedback and polar warming amplification. Part II: meridional and vertical asymmetries of the global warming. Clim. Dyn..

[76] Feldl N, Roe GH (2013). The nonlinear and nonlocal nature of climate feedbacks. J. Clim..

[77] Zelinka MD, Hartmann DL (2011). Climate feedbacks and their implications for poleward energy flux changes in a warming climate. J. Clim..

[78] Overland JE (2016). Nonlinear response of mid-latitude weather to the changing Arctic. Nat. Clim. Change.

[79] Marshall J (2015). The ocean’s role in the transient response of climate to abrupt greenhouse gas forcing. Clim. Dyn..

[80] Jungclaus JH, Lohmann K, Zanchettin D (2014). Enhanced 20th-century heat transfer to the Arctic simulated in the context of climate variations over the last millennium. Clim. Past..

[81] Bitz CM, Gent PR, Woodgate RA, Holland MM, Lindsay R (2006). The influence of sea ice on ocean heat uptake in response to increasing CO_2_. J. Clim..

[82] Gregory JM (2004). A new method for diagnosing radiative forcing and climate sensitivity. Geophys. Res. Lett..

[83] Taylor KE (2007). Estimating shortwave radiative forcing and response in climate models. J. Clim..

[84] Soden BF (2008). Quantifying climate feedbacks using radiative kernels. J. Clim..

[85] Shell KM, Kiehl JT, Shields CA (2008). Using the radiative kernel technique to calculate climate feedbacks in NCAR’s community atmospheric model. J. Clim..

[86] Jonko A, Shell K, Sanderson B, Danabasoglu G (2012). Climate feedbacks in CCSM3 under changing CO_2_ forcing. Part I: adapting the linear radiative kernel technique to feedback calculations for a broad range of forcings. J. Clim..

[87] Jonko AK, Shell KM, Sanderson BM, Danabasoglu G (2013). Climate feedbacks in CCSM3 under changing CO_2_ forcing: Part II. Variation of climate feedbacks and sensitivity with forcing. J. Clim..

[88] Sedlar J, Shupe MD, Tjernström M (2012). On the relationship between thermodynamic structure and cloud top, and its climate significance in the Arctic. J. Clim..

[89] Cai M, Lu J (2009). A new framework for isolating individual feedback processes in coupled general circulation climate models. Part II: method demonstrations and comparisons. Clim. Dyn..

[90] Feldl N, Roe GH (2013). Four perspectives on climate feedbacks. Geophys. Res. Lett..

[91] Sherwood SC (2015). Adjustments in the forcing-feedback framework for understanding climate change. Bull. Am. Meteor. Soc..

[92] Andrews T, Gregory JM, Webb MJ (2015). The dependence of radiative forcing and feedback on evolving patterns of surface temperature change in climate models. J. Clim..

[93] Colman RA, McAvaney BJ (2011). On tropospheric adjustment to forcing and climate feedbacks. Clim. Dyn..

[94] Meraner K, Mauritsen T, Voigt TA (2013). Robust increase in equilibrium climate sensitivity under global warming. Geophys. Res. Lett..

[95] Gregory JM, Andrews T, Good P (2015). The inconstancy of the transient climate response parameter under increasing CO_2_. Philos. Trans. Roy. Soc. Lond..

[96] Rugenstein MAA, Caldeira K, Knutti R (2016). Dependence of global radiative feedbacks on evolving patterns of surface heat fluxes. Geophys. Res. Lett..

[97] Holland MM, Bitz CM (2003). Polar amplification of climate change in coupled models. Clim. Dyn..

[98] Sejas SA (2014). Individual feedback contributions to the seasonality of surface warming. J. Clim..

[99] Svensson G, Karlsson J (2011). On the Arctic wintertime climate in global climate models. J. Clim..

[100] de Boer G, Chapman W, Kay JE, Medeiros B, Shupe MD (2012). A characterization of the present-day Arctic atmosphere in CCSM4. J. Clim..

[101] Qu X, Hall A (2007). What controls the strength of snow-albedo feedback?. J. Clim..

[102] Notz D (2016). The CMIP6 Sea–Ice Model Intercomparison Project (SIMIP): understanding sea ice through climate-model simulations. Geosci. Model Dev..

[103] Pithan F (2016). Select strengths and biases of models in representing the Arctic winter boundary layer over sea ice: the Larcform 1 single column model intercomparison. J. Adv. Mod. Earth Syst..

[104] Hawkins E, Sutton R (2016). Connecting climate model projections of global temperature change with the real World. Bull. Am. Meteor. Soc..

[105] Knutti R (2008). Why are climate models reproducing the observed global surface warming so well?. Geophys. Res. Lett..

[106] Bitz, C. M. Some aspects of uncertainty in predicting sea ice thinning. Arctic sea ice decline: observations, projections, mechanisms, and implications, *Amer. Geophys. Union* (eds deWeaver, E., Bitz, C. M. & Tremblay, B.) 63–76 (2008).

[107] Meehl GA, Washington WM (2013). Climate change projections in CESM1(CAM5) compared to CCSM4. J. Clim..

[108] Wang M, Overland JE (2012). A sea ice free summer Arctic within 30 years: an update from CMIP5 models. Geophys. Res. Lett..

[109] Massonnet F (2012). Constraining projections of summer Arctic sea ice. Cryosphere.

[110] Eyring V (2016). Towards improved and more routine Earth system model evaluation in CMIP. Earth Syst. Dynam..

[111] Gloersen, P. et al. Arctic and Antarctic sea ice, 1978–1987: satellite passive microwave observations and analysis. National Aeronautics and Space Administration, NASA SP-511 (1992).

[112] Barthélemy A, Fichefet T, Goosse H, Madec G (2015). Modeling the interplay between sea ice formation and the oceanic mixed layer: limitations of simple brine rejection parameterizations. Ocean Model..

[113] Gent PR (2011). The community climate system model version 4. J. Clim..

[114] Dufresne JL (2013). Climate change projections using the IPSL-CM5 earth system model: from CMIP3 to CMIP. Clim. Dyn..

[115] Ridgway KR, Dunn JR, Wilkin JL (2002). Ocean interpolation by four-dimensional weighted least squares-application to the waters around Australasia. J. Atmos. Ocean. Tech..

[116] Massonnet F (2013). A model reconstruction of the Antarctic sea ice thickness and volume changes over 1980–2008 using data assimilation. Ocean Model..

[117] Donohoe A, Armour KC, Pendergrass AG, Battisti DS (2014). Shortwave and longwave radiative contributions to global warming under increasing CO_2_. Proc. Nat. Acad. Sci..

