# Supplementary material for: Quantifying climate feedbacks in polar regions
Source: Nat Commun. 2018 May 15;9:1919. doi: 10.1038/s41467-018-04173-0 (PMC5953926; doi:10.1038/s41467-018-04173-0)
Supplement: Supplementary file 1 — Supplementary Information [file 41467_2018_4173_MOESM1_ESM.pdf]

Quantifying climate feedbacks in polar regions

Supplementary Material

Hugues Goosse[1], Jennifer E. Kay[2], Kyle C. Armour[3], Alejandro Bodas-Salcedo[4], Helene Chepfer[5], David Docquier[1], Alexandra Jonko[6], Paul J. Kushner[7], Olivier Lecomte[1], François Massonnet[1,8], Hyo-Seok Park[9], Felix Pithan[10], Gunilla Svensson[11], Martin Vancoppenolle[12]

1. Earth and Life Institute, Université catholique de Louvain, Belgium
2. Department of Atmospheric and Oceanic Sciences, and Cooperative Institute for Research in Environmental Science, University of Colorado – Boulder, USA
3. School of Oceanography and Department of Atmospheric Sciences, University of Washington, Seattle, USA
4. Met Office Hadley Centre, Exeter, United Kingdom.
5. Sorbonne Université, UPMC Paris 6, LMD-IPSL, CNRS, France
6. Earth and Environmental Sciences Division, Los Alamos National Laboratory, USA
7. Department of Physics, University of Toronto, Canada
8. Earth Sciences Department, Barcelona Supercomputing Center, Barcelona, Spain.
9. Korea Institute of Geoscience and Mineral Resources, Daejeon, South Korea
10. Alfred Wegener Institute, Helmholtz Centre for Polar and Marine Research, Bremerhaven, Germany
11. Department of Meteorology and Bolin Center for Climate Research, Stockholm University, Sweden
12. Sorbonne Universités, UPMC Paris 6, LOCEAN-IPSL, CNRS, France

Contact author: Hugues Goosse, Earth and Life Institute, Université catholique de Louvain, Place Pasteur 3, Louvain-la-Neuve, Belgium. Email: hugues.goosse@uclouvain.be

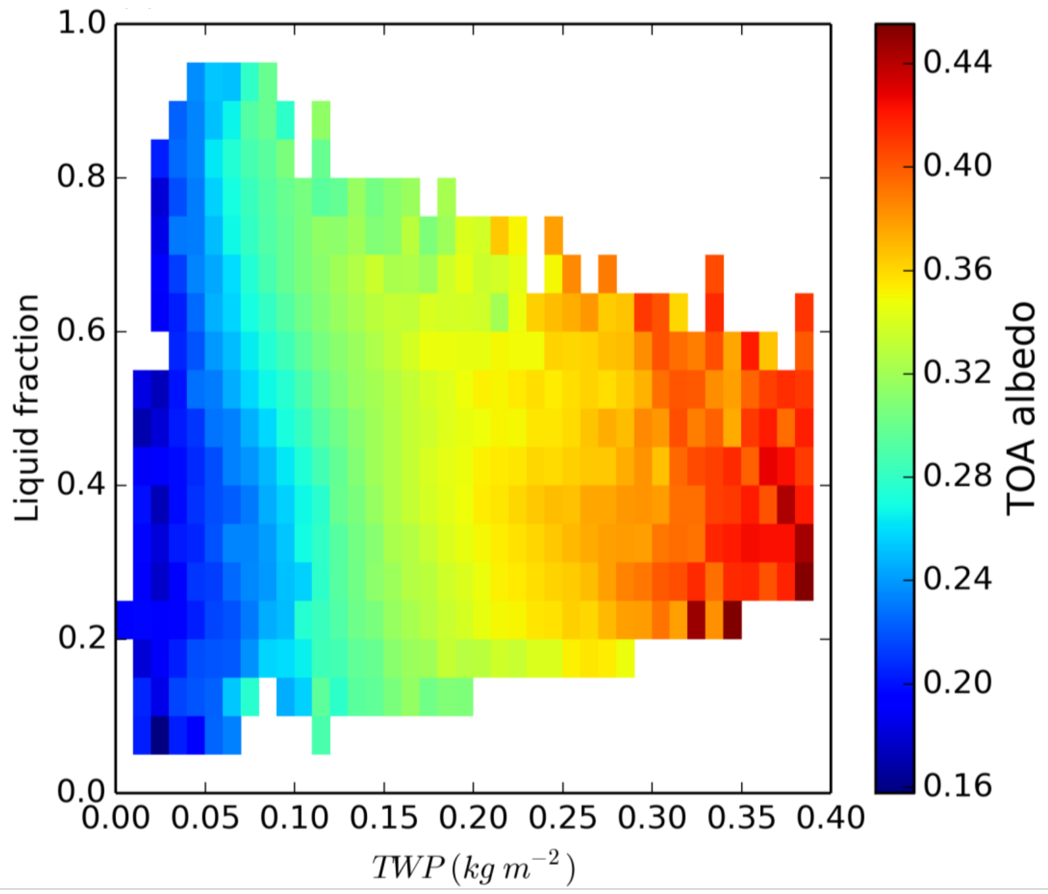

Supplementary Figure 1. Dependency of top of the atmosphere (TOA) albedo (fraction of solar radiation that is reflected back to space) with cloud liquid fraction and total water path (TWP, corresponding the total mass of cloud water per unit area). It is calculated using monthly mean data for December to February, restricted to the latitudes between 50 and 60 degrees south. Data are from an atmosphere-only simulation of the Hadley Centre Global Environmental Model version 2.

Supplementary Note 1. Comparison of different evaluation methods of the feedback factor γ for the ice production-oceanic entrainment feedback.

The feedback factor γ_θ for the ice production-ocean entrainment feedback can be related to the feedback gain *G*, defined as the ratio between the changes in the system if the feedback is active (Δh) on the changes if the feedback is not active (Δh_0).

$$G = \frac{\Delta h}{\Delta h_0} \quad \text{(S1)}$$

γ_θ can also be estimated as originally proposed[1] by only considering the initial melt due to entrainment, not the equilibrium response, i.e. considering that the ice formation induces entrainment of warmer water and thus ice melting but that the additional consequences of this melting are not accounted for yet. This is actually the most straightforward way to estimate its value from observed profiles (see also Supplementary Note 3).

Consider first that because of heat loss at the surface, an amount of ice Δh_0 can be formed (reference response). That would be the amount formed on a lake at freezing point temperature, for instance, i.e. without the ice production-entrainment feedback. In the Southern Ocean, the ice production-entrainment feedback induces an initial melting of Δh_r ($\Delta h_r < 0$) in response to the formation Δh_0. The total thickness change after this first step is then

$$\Delta h_1 = \Delta h_0 + \Delta h_r \quad \text{(S2)}$$

If one defines γ as :

$$\gamma = \frac{\textit{Initial melt due to entrainment}}{\textit{Initial ice formation}} = \frac{\Delta h_r}{\Delta h_0} \quad \text{(S3)}$$

One obtains

$$\Delta h_1 = \Delta h_0 + \Delta h_r = (1+\gamma)\Delta h_0 \qquad \text{(S4)}$$

Because of the feedback, ice formation is thus modified by $\gamma\,\Delta h_0$. The impact of the entrainment feedback on this additional perturbation is $\gamma^2 \Delta h_0$, and so on. The series converges (for $|\gamma|$ <1) to (see for instance ref. 2):

$$\Delta h = (1+\gamma+\gamma^2+\gamma^3+\ldots)\Delta h_0 = \tfrac{1}{1-\gamma}\Delta h_0 \qquad \text{(S5)}$$

and the gain G is :

$$G = \tfrac{1}{1-\gamma} \qquad \text{(S6)}$$

The gain can also be directly calculated (i.e. without assuming several artificial feedback loops and the convergence of the series) by considering that the feedback acts finally on the total ice thickness change and thus at equilibrium

$$\Delta h = \Delta h_0 + \gamma\,\Delta h \qquad \text{(S7)}$$

Leading directly to:

$$G = \frac{\Delta h}{\Delta h_0} = \frac{1}{1-\gamma} \qquad \text{(S8)}$$

The definition of γ is thus the same as γ_θ in the main body of the paper since :

$$1-\gamma = \frac{\Delta h_0}{\Delta h} \qquad \text{(S9)}$$

and thus

$$\gamma = \frac{\Delta h - \Delta h_0}{\Delta h} = \gamma_\theta \qquad \text{(S10)}$$

Supplementary Note 2. Evaluation of the feedback factor γ for the ice growth-ice thickness feedback.

Using some reasonable assumptions, the ice production-entrainment feedback can be calculated analytically from existing oceanic profiles, both from observations[1] and model outputs. Unfortunately, analytical calculations are often not possible and, as is the case for radiative feedbacks, specific additional computations can be required to estimate the factor γ for some non-radiative feedbacks. This is illustrated here for the ice growth-thickness feedback[3,4] considering the classical Semtner 0-layer approach for sea ice thermodynamics[5]. This model combines a surface energy balance and simplified heat conduction-driven growth rate that can be used to compute the seasonal and inter-annual evolution of sea ice thickness. However, it neglects ice dynamics and heat storage within the snow and sea ice. As a consequence, the magnitude of the feedback factor estimated from this model is likely strongly biased but we present it here to show how the formalism proposed in the main text can be applied when additional numerical experiments are necessary.

To estimate a feedback factor γ_{k} associated with this feedback, we consider 1) a perturbation of the surface energy budget, 2) the sea ice thickness h as the key variable, 3) the total system as the sea ice cover with thickness dependence on growth rate, and 4) the reference system as the sea ice cover without thickness dependence on growth rate. Consequently, we define Δh as the equilibrium ice thickness change of the full system due to the perturbation, and Δh_0 as the equilibrium ice thickness response that would be obtained for the same perturbation if the thickness-dependence of ice growth rates is omitted (i.e., growth independent of thickness).

A definition in line with the general framework described here (Eq. 1 of the main text) is then given by the following feedback factor:

$$\gamma_k = \frac{\Delta h - \Delta h_0}{\Delta h} \quad \text{(S13)}$$

Δh and Δh_0 are estimated by integrating the model subject to a radiative perturbation, with or without letting the growth rates depend on the sea ice thickness. The curves in Supplementary Figure 2 are based on ~10 series of simulations, each corresponding to a given baseline energetic forcing level, giving a [1.5 m] range in the initial equilibrium thickness. Each of the 10 series includes 3 simulations: a control run and two perturbed runs with +1 W/m^2 applied to the surface energy budget. The first perturbed run has all feedbacks enabled, which gives the basis to compute Δh (black line in Supplementary Figure 2). In the second run, used to compute Δh_0, the thickness dependency of the ice growth rate is switched off, using the seasonal cycle of ice thickness from the control run instead of the online thickness (blue line in Supplementary Figure 2).

Following a positive perturbation of the surface energy budget, the decrease in sea ice thickness is smaller with the feedback (Δh) than without it (Δh_0) (Supplementary Figure 2, left). The feedback is nonlinear because the conduction flux is inversely proportional to the sea ice thickness. When the feedback is active, a slight reduction in sea ice thickness is sufficient to induce a larger winter growth rate that compensates for the additional melt rate imposed by the radiative perturbation. Those thickness changes are smaller for thin ice (in absolute value) thanks to the dependence of growth rates on the inverse of sea ice thickness[3,4]. In contrast, when the dependence of the growth rate on thickness is turned off, the equilibrium balance between annual growth and melt is attained through the reduction in summer melt. This is due to a decrease in summertime downward heat conduction with ice thickness, inducing a reduction of the energy available for melting ice as the ice thins. Since the associated changes in surface heat budget are relatively weak, achieving the annual growth-melt balance requires a much larger thickness reduction without the feedback than when it is active.

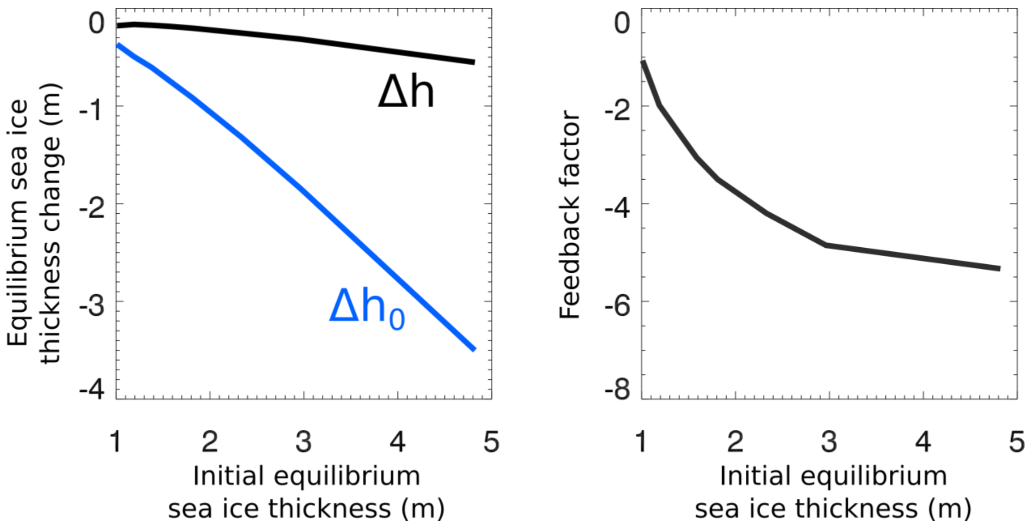

Supplementary Figure 2. Computation of the sea ice growth-thickness feedback factor in the Semtner 0-layer model[5]. (Left) Difference in maximum sea ice thickness between a control and a perturbed simulation with (Δh) and without (Δh_0) the ice growth ice-thickness feedback. (Right) Feedback factor γ_k corresponding to the sea ice growth-thickness feedback. All values are plotted versus the initial equilibrium thickness to highlight the mean-state dependence of the response.

Supplementary Note 3: a simple model for the ice production-entrainment feedback

The non-linearity of the ice production-entrainment feedback can be described using a simplified representation of the water column in the Southern Ocean. Consider a mixed layer below sea ice that is at the freezing point T_f as long as sea ice is present (Supplementary Figure 3). The mixed layer has initially a salinity S_0. Below the mixed layer (i.e., in the pycnocline), the temperature and salinity are increasing in several staircases of the same magnitude. When the brine release due to ice formation is able to increase the salinity to the value of the next staircase, the water column becomes unstable and pycnocline water is entrained in the mixed layer (actually, as the pycnocline is warmer than the mixed layer, the destabilization occurs slightly before the salinity of the mixed layer reached the one of the pycnocline but this is

neglected here). Following the nomenclature of ref. 1, the salt input due to ice formation required to destabilize the water column is called the salt deficit (SD_w) and is equal here to:

$$SD_\mathrm{w} = \frac{\rho_\mathrm{i} L_\mathrm{i}}{\left(S_n - S_i\right)}\left(S_n - S_{n\text{-}1}\right)\left(h_\mathrm{wm} + n\Delta h_\mathrm{wp}\right) \tag{S11}$$

where S_n is the salinity of the layer n of the 'staircase', h_wm the mixed layer depth before the destabilization and Δh_wp the depth of each 'staircase'. ρ_i, L_i and S_i are the sea ice density, latent heat of fusion and salinity, respectively.

The heat input associated to the entrainment (TB_w the winter thermal barrier in Martinson's terminology) is

$$TB_\mathrm{w} = \rho_\mathrm{w} c_\mathrm{w} \left(T_n - T_\mathrm{f}\right)\Delta h_\mathrm{wp} \tag{S12}$$

where ρ_w and c_w are the water density and heat capacity, respectively, and T_n is the temperature of the 'staircase' n.

The coefficient $\rho_\mathrm{i} L_\mathrm{i} / \left(S_\mathrm{n} - S_\mathrm{i}\right)$ has been introduced in Eq. (S11) so that SD_w corresponds to the energy needed to melt the sea ice and $\gamma = TB_\mathrm{w} / SD_\mathrm{w}$ is dimensionless and corresponds to the ratio between the melt due to entrainment and the initial ice formation. It is thus equivalent to the definition of γ_θ in the main body of the paper and in Supplementary Note 1. The development also illustrates that γ can be evaluated from temperature and salinity profiles only, as originally proposed[1].

It is clear from (S11) that for a steady sea ice production, SD_w remains constant for each staircase as the mixed layer salinity increases to be always equal to the one of the pycnocline water that have just been entrained in the mixed layer. This is different for temperature. Because the mixed layer is constrained to remain close to the freezing point by the presence of sea ice,

the difference between the temperature of the mixed layer and the one of the pycnocline increases as the mixed layer deepens, leading to an increase in the magnitude of γ_θ.

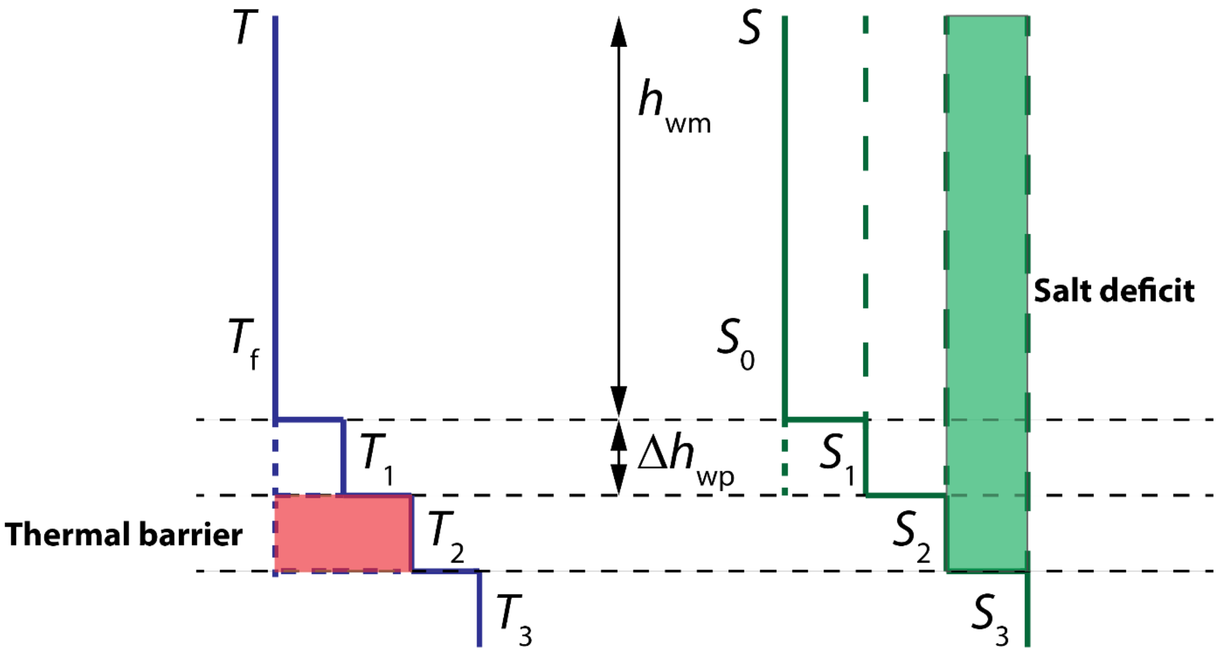

Supplementary Figure 3. Simplified oceanic profiles illustrating the ice production-entrainment feedback.

References

1. Martinson, D. G. Evolution of the Southern Ocean winter mixed layer and sea ice-open ocean deep-water formation and ventilation. *J. Geophys. Res. Oceans* **95**, 11641-11654 (1990).
2. Gregory, J.M., Jones, C.D., Cadule, P. & Friedlingstein, P. Quantifying carbon cycle feedbacks. *J. Climate* **22**, 5232-5250 (2009).
3. Maykut, G.A. The surface heat and mass balance. *The Geophysics of Sea Ice*, Untersteiner, N., Ed., Plenum Press, pp. 395–464 (1986).
4. Bitz, C. M. & Roe, G. H. A mechanism for the high rate of sea ice thinning in the Arctic Ocean. *J. Climate* **17**, 3623-3632 (2004).
5. Semtner Jr., A.J. A model for the thermodynamic growth of sea ice in numerical investigations of climate. *J. Phys. Oceanogr.* **6**, 379– 389 (1976).
